# Use of an Acellular Regenerative Tissue Matrix Over Chronic Wounds

**Published:** 2013-11-20

**Authors:** D. Heath Stacey

**Affiliations:** Northwest Arkansas Center for Plastic Surgery, Fayetteville, Ark

**Keywords:** chronic wound, human acellular dermal matrix, lower extremity, matrix, NPWT

## Abstract

**Objectives:** Bioengineered skin grafts, including acellular dermal matrices, may be effective in treating lower extremity and trunk wounds that are not responsive to traditional wound management. Acellular dermal wound matrix is derived from human acellular dermal wound matrix (HADWM) tissue and provides a scaffold that supports cellular repopulation and revascularization. The major structural components of the dermis are retained during processing, and a single application has been shown to help achieve wound closure. **Methods:** This patient case series examined the use of HADWM on lower extremity and trunk wounds in 11 patients (6 male and 5 female) with a mean age of 55 years (range: 31–83 years). Wounds were debrided 1 to 2 times, followed by placement of HADWM (range: 4–330 cm^2^) on wounds that varied from the dorsal surface of the foot, lower abdomen, and lower extremity to the Achilles flap. A nonadherent layer in conjunction with bacitracin was placed over HADWM. Negative pressure wound therapy (NPWT) was placed over the HADWM and initiated continuously at −125 mm Hg for 1 to 2 weeks. After the application of NPWT, HADWM was covered with various gauze dressings using mineral oil. **Results:** All patients completed their treatment successfully, and follow-up ranged from 1 week to 6 months. One patient experienced an infection, which resulted in partial graft loss that required replacement with HADWM and NPWT. No additional complications occurred in the other patients. **Conclusions:** This patient case series demonstrated successful use of HADWM and NPWT, which further supports published studies documenting HADWM success in chronic wounds.

Wounds of the lower extremity currently affect more than 6 million people in the United States.^1^ Chronic full-thickness wounds, such as lower extremity wounds, can be hard to heal and can become a problem to treat.^2^ Nonhealing or slow healing wounds create a major health problem that contributes to substantial disability, morbidity, and costs. Currently, there is a need to develop treatment modalities to reduce the risks of severe extremity wounds that can lead to amputations.^3^

Bioengineered skin grafts, including acellular dermal matrices, may be effective in treating lower extremity wounds that are not responsive to traditional wound management.^4^ In order for skin substitutes to be effective, the wound bed must be well vascularized and debrided to healthy tissue.^5^ Tissue engineering research has shown benefits to using allografts to reach faster wound healing as compared with conservative care.^6^ Also, several studies have shown the successful use of human acellular dermal regenerative matrices to heal chronic, full-thickness wounds of the lower extremity.^2^^,^^6^

When using the appropriate acellular matrix, wounds may heal faster than standard treatment in chronic hard-to-heal wounds.^4^ Acellular dermal matrices may be used to replace damaged extracellular matrix, fill defects, and optimize the wound environment for healing lower extremity wounds.^7^ By combining an acellular matrix with other advanced treatment modalities, wounds may continue the progression of healing.^7^ For example, the use of negative pressure wound therapy (NPWT, V.A.C. Therapy, KCI USA, Inc, San Antonio, Texas) may help to control excessive exudate and, in addition, hold the matrix in place to maximize contact with the wound bed.^8^

Graftjacket regenerative tissue matrix (Wright Medical Technology, Inc, licensed by KCI USA, Inc, San Antonio, Texas) is a human acellular dermal wound matrix (HADWM) that is derived from human tissue and processed from screened donated human skin.^4^ The HADWM is processed to remove the living cells while preserving dermal structure^4^ and also serves as a scaffold to support cellular repopulation and revascularization.^4^ Wound bed preparation is crucial and requires debridement of necrotic tissue and control of infection and edema.^9^ This article reports clinical experience using HADWM in conjunction with NPWT on patients with lower extremity and trunk wounds.

## METHODS

Each patient underwent a complete medical history, a physical examination, and full assessment of the wound. Informed consent was obtained for each patient before treatment.

All chronic wounds were debrided 1 to 2 times to remove necrotic tissue. After sharp debridement, a bleeding wound base was created, followed by placement of HADWM (range: 4–330 cm^2^).

If needed, multiple HADWMs were combined to completely cover the surface area of larger wounds. The reticular layer (shiny side) of the HADWM was placed against the wound bed, which aligned this layer to the vascular supply of adjacent tissue and the basement membrane. HADWM was secured with staples to the wound margins to ensure coverage of the whole wound bed. A nonadherent layer (Adaptic, Systagenix Wound Management, Gatwick, UK) in conjunction with bacitracin was placed over the HADWM.

Negative pressure wound therapy was used as a bolster over the HADWM, and pressure was initiated continuously at −125 mm Hg for 1 to 2 weeks. After NPWT application, HADWM was covered with various gauze dressings moistened with mineral oil. Dressings were changed and reapplied every 1 to 2 days.

## RESULTS

All patients completed their treatment successfully, and follow-up ranged from 1 week to 6 months. Patient demographics are summarized in Table 1. A total of 11 patients (6 male and 5 female) received HADWM to treat lower extremity and trunk wounds followed by NPWT. The mean patient age was 55 years, with patients ranging in age from 31 to 83 years. Patient comorbidities are listed in Table 2. Two patients experienced an infection; one resulted in partial graft loss that required replacement of HADWM and NPWT. Both patients’ wounds healed completely. All wounds reached complete healing with 10 of 11 patient wounds receiving a single application of HADWM. No complications specifically associated with the use of the HADWM were noted.

## CASE STUDIES

**Figure 1:**
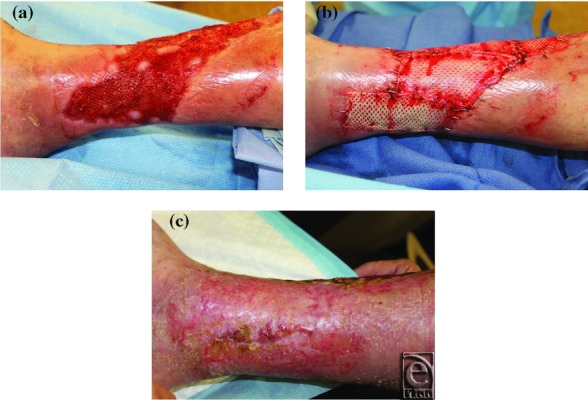
(*a*) Wound at initial presentation. (*b*) Wound after HADWM placement. (*c*) 30 days after placement of HADWM.

**Figure 2:**
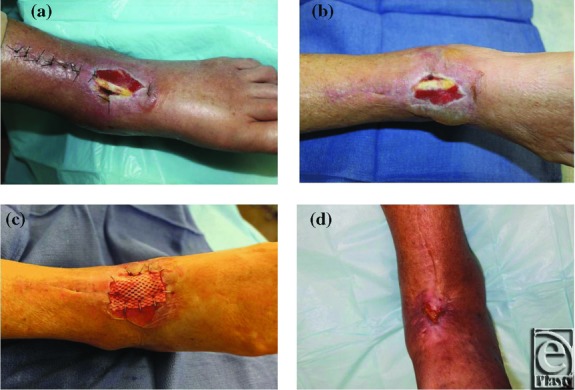
(*a*) Wound at initial presentation. (*b*) Sutures removed. (*c*) Wound after HADWM placement. (*d*) Approximately 6 months after HADWM placement.

**Figure 3:**
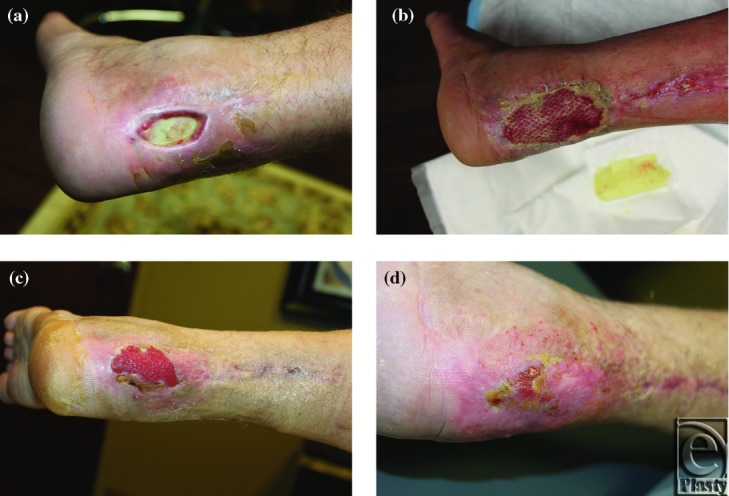
(*a*) Wound at initial presentation. (*b*) Wound after HADWM placement. (*c*) 8 days after HADWM placement. (*d*) 45 days after HADWM placement.

Three cases are highlighted in Figures 1 to 3 and demonstrate the successful use of HADWM in conjunction with NPWT.

### Patient 1

A 72-year-old man with history of venous insufficiency ulcers in both legs with a large, chronic, lower extremity wound (Figures 1a-c).

### Patient 2

An 83-year-old woman with a postsurgical right dorsal foot wound after a total ankle arthroplasty (Figures 2a-d).

### Patient 3

A 39-year-old man with chronic postsurgical Achilles wound with exposed tendon and scar tissue (Figures 3a-d).

## DISCUSSION

The use of HADWM in conjunction with NPWT resulted in complete healing in all of the lower extremity and trunk wounds in this series of patients. These results further support previous findings of the successful use of acellular matrices used and in combination with NPWT.^2^^,^^3^^,^^8^^,^^10^^,^^11^

Negative pressure wound therapy has been shown to increase perfusion, contraction, and rate of granulation in acute and chronic wounds.^5^ By using NPWT in conjunction with HADWM, the wound is kept moist, thus enhancing its integration. Negative pressure wound therapy also acts as a bolster and further secures the HADWM to the wound bed. The use of NPWT with HADWM may enhance the adherence and survival of matrices. Several clinical studies have shown the successful use of NPWT in managing matrices.^12^^-^16

Human acellular dermal wound matrix has several advantages over other bioengineered skin grafts.^4^ For instance, HADWM is derived from human cadaver tissue and composed of collagen and extracellular protein matrices. These proteins promote nutritional diffusion and cellular proliferation at the graft site, leading to rapid revascularization and cellular repopulation of the matrix. The scaffold then transitions into essential dermal tissue, which further reproduces its own tissues around it. The patients in this series have regenerated tissue that is more functionally stable than scar tissue. Another benefit of HADWM is the avoidance of any donor site, which may be problematic in a patient who is immunocompromised or has other comorbidities that may lead to a healing delay.

A single application of HADWM is often sufficient for complete healing and may potentially translate into a further cost saving when compared with other bioengineered grafts that require multiple treatments. Other studies have shown that multiple applications of dermal matrices may be required to heal chronic wounds.^17^^,^^18^ In addition, there is a cost to the patient in terms of lost days of work and longer time to healing. Benefits of combination therapy, such as HADWM and NPWT, are that the procedure can be performed in a clinical setting, further avoiding cost of surgery and preoperative testing as well as eliminating the need for daily dressing changes. Our initial experience further supports evidence that skin grafts, such as HADWM used in conjunction with NPWT, are effective treatment options for lower extremity wounds. While our experience was limited to 11 patients, larger studies with defined endpoints (eg, time to wound closure) are needed to confirm our results of this combination therapy.

## Figures and Tables

**Table 1:** Patient demographics

Patient no.	Sex	Age	Comorbidities	Wound etiology	Wound location	Prior treatments	Human Acellular Dermal Matrix size	Bolster
1	F	83	Diabetes mellitus, hypertension, osteoarthritis, heart disease, vascular insufficiency	Postsurgical, vascular insufficiency to the foot	Right dorsal foot	Debridement by orthopedic surgeon, attempted closure	4 cm^2^	NPWT
2	M	53	Venous insufficiency, morbid obesity	Venous stasis ulcer, lymphedema skin breakdown	Lower extremity	Compression therapy, non-adherent dressing changes daily	40 cm^2^	NPWT
3	F	52	Uterine cancer, smoking, thyroid cancer	Failed full-thickness skin graft, exposed tendon	Right dorsal foot	Negative pressure wound therapy	4 cm^2^	NPWT
4	M	72	Venous insufficiency	Multiple debridements of venous stasis ulcers	Right lower extremity	Compression therapy, Apligraf®	330 cm^2^	NPWT
5	M	39	None	Postsurgical wound after Achilles repair	Right Achilles adipofascial flap	Wet to dry dressings	32 cm^2^	NPWT
6	F	31	Diabetes mellitus, kidney disease, liver failure	Venous stasis and posttraumatic wound	Right lower extremity	Wet to dry dressings	120 cm^2^	NPWT
7	M	64	Diabetes mellitus, Fournier gangrene	Debridements of necrotic tissue	Lower left abdomen	Negative pressure wound therapy	32 cm^2^	NPWT
8	M	54	Diabetes mellitus	Debridement of pressure ulcers	Sacrum ulcer, Diabetic ulcer on left foot, distal forefoot ulcer on right foot, exposed toe bone on dorsum of foot, and large ulcer on midfoot	Wet to dry dressings	64 cm^2^	NPWT
9	M	65	Diabetes mellitus, hypertension, Fournier gangrene, coronary heart disease, renal disease	Debridement of pressure ulcers	Bilateral calcaneal ulcers	Wet to dry dressings	64 cm^2^	NPWT
10	F	53	Uterine cancer, smoking, thyroid cancer	Postsurgical	Right dorsal foot	Wet to dry dressings	4 cm^2^	NPWT
11	F	34	Smoking	Posttraumatic	Left	Negative pressure wound therapy followed by wet to dry dressings	150 cm^2^	NPWT
